# Massive Esophageal Dilatation Following Laparoscopic Adjustable Gastric Banding Presenting as Acute Respiratory Distress After Swallowing Effervescent Granules: A Case Report

**DOI:** 10.7759/cureus.116327

**Published:** 2026-09-15

**Authors:** Amanda Lu, Yung-Hsien Wang, Saikiran Jonnadula

**Affiliations:** 1 Hospital Medicine, Brigham and Women's Hospital, Harvard Medical School, Boston, USA; 2 Medicine, Brigham and Women's Hospital, Harvard Medical School, Boston, USA

**Keywords:** airway compression, esophageal dilatation, laparoscopic adjustable gastric banding, left atrium compression, megaesophagus

## Abstract

Esophageal dilatation is a recognized late complication of laparoscopic adjustable gastric banding (LAGB), resulting from chronic outflow obstruction and esophageal dysmotility. Although most cases are clinically mild, severe dilatation can produce mass effect on adjacent mediastinal structures and rarely cause acute cardiopulmonary compromise.

A 63-year-old female with a history of obesity, hypertension, glaucoma, and LAGB placement more than 15 years earlier presented with acute respiratory distress during an upper gastrointestinal contrast study. Shortly after swallowing effervescent granules, she developed abrupt chest pressure, dyspnea, wheezing, cough, nausea, and abdominal bloating. A resuscitation team was activated because of concerns for acute respiratory distress, and she was empirically treated for anaphylaxis with intramuscular epinephrine, corticosteroids, antihistamines, nebulized epinephrine, bronchodilators, and supplemental oxygen. Her symptoms rapidly resolved. However, she had no urticaria, angioedema, hypotension, or persistent bronchospasm. Imaging demonstrated a markedly dilated, gas-filled esophagus measuring up to 75 mm in diameter. Blood gas analysis during the episode demonstrated acute respiratory acidosis with hypercapnia, which subsequently normalized.

The patient's acute respiratory distress was attributed to sudden gaseous expansion of a chronically dilated esophagus following administration of effervescent granules, resulting in transient airway compression. This case highlights a rare but potentially life-threatening manifestation of severe esophageal dilatation following LAGB and emphasizes the importance of considering structural causes of acute respiratory distress when the clinical features of anaphylaxis are absent.

## Introduction

Laparoscopic adjustable gastric banding (LAGB) was historically one of the most commonly performed bariatric procedures because of its reversibility and relative technical simplicity. Although its use has declined substantially, many patients continue to live with gastric bands placed years or decades earlier. Long-term complications include band slippage, pouch enlargement, gastroesophageal reflux, esophageal dysmotility, and progressive esophageal dilatation.

Esophageal dilatation following LAGB is thought to result from chronic functional outflow obstruction at the gastroesophageal junction. Excessive band restriction can increase resistance to esophageal emptying, leading over time to progressive esophageal enlargement and impaired peristalsis, with an “achalasia-like” pattern of impaired esophageal clearance despite the absence of primary achalasia. Reported frequencies of esophageal abnormalities after LAGB vary widely, in part because studies have used different definitions of radiographic dilatation and esophageal dysmotility, as well as different durations of follow-up [1,2]. Thus, radiographic esophageal dilatation, objective dysmotility, and severe symptomatic megaesophagus should be regarded as distinct clinical entities rather than interchangeable manifestations.

While many patients with post-LAGB esophageal dilatation can be managed by adjustment or deflation of the band, progressive enlargement can become clinically significant when the esophagus occupies substantial mediastinal space and exerts mass effect on adjacent structures. Reported manifestations include dysphagia, regurgitation, nocturnal cough, wheezing, dyspnea, aspiration, and, rarely, airway or cardiovascular compression [3,4]. Massive esophageal dilatation with acute respiratory compromise is exceptionally uncommon and may present with features that mimic other causes of sudden respiratory distress.

We present a case of massive esophageal dilatation measuring 75 mm in maximal diameter in a patient more than 15 years after LAGB placement. Following ingestion of an effervescent preparation, the patient developed abrupt respiratory distress, initially raising concern for anaphylaxis. Although contemporaneous dynamic evidence was not available to establish the mechanism, the temporal association and subsequent clinical course suggested that acute gaseous expansion of the markedly dilated esophagus may have exacerbated pre-existing mediastinal mass effect. This case highlights a rare acute presentation of longstanding LAGB-associated esophageal disease and emphasizes the importance of considering acute esophageal expansion as a potential contributor to respiratory compromise in patients with severe esophageal dilatation.

## Case presentation

A 63-year-old woman with a history of obesity, hypertension, glaucoma, and LAGB performed more than 15 years earlier was transferred to the emergency department from an outpatient radiology suite because of acute respiratory distress during a scheduled upper gastrointestinal contrast examination. The examination was being performed as part of the evaluation of longstanding esophageal symptoms.

During the examination, the patient swallowed a pack of effervescent crystals followed by small amounts of water. The patient reported having undergone similar examinations previously, during which the effervescent preparation had reportedly been dissolved in water rather than swallowed as granules.

Within moments of swallowing the effervescent preparation, she developed an abrupt sensation described as an “explosion” in her chest, followed by severe chest pressure, dyspnea, wheezing, cough, nausea, and a sensation of chest and abdominal bloating. Venous blood gas analysis obtained during the episode demonstrated acute respiratory acidosis with hypercapnia and hypoxemia (pH 7.29, partial pressure of carbon dioxide (PCO_2_) 61 mmHg, partial pressure of oxygen (PO_2_) 45 mmHg, and mixed venous oxygen saturation (SvO_2_) 73.2%). A respiratory response was activated in the radiology suite.

Because of the acute respiratory symptoms and wheezing, anaphylaxis was considered and treated empirically. She received two 0.3-mg intramuscular doses of epinephrine, intravenous methylprednisolone 100 mg, intravenous diphenhydramine 50 mg, famotidine, nebulized epinephrine, repeated nebulized bronchodilators, and supplemental oxygen. She initially required high-flow nasal cannula oxygen at 50% inspired oxygen and a flow rate of 50 L/min. Her respiratory status subsequently improved, and by arrival in the emergency department she was speaking comfortably in full sentences without apparent respiratory distress.

On emergency department evaluation, her blood pressure was 202/80 mmHg and her heart rate was approximately 90-100 beats/min. Oxygen saturation was normal on room air. She was afebrile and in no acute distress. Repeated venous blood gas analysis demonstrated resolved hypercapnic acidosis (pH 7.36, PCO_2_ 48 mmHg, PO_2_ 38 mmHg, SvO_2_ 68.8%). Cardiac examination demonstrated a regular rhythm, and the lungs were clear to auscultation without persistent wheezing. There was no urticaria, angioedema, rash, or peripheral edema. These findings describe the examination after treatment and therefore do not exclude transient bronchospasm, anaphylaxis, aspiration, or another acute airway process at the time of symptom onset.

Her history was notable for several years of progressive upper gastrointestinal and respiratory symptoms, including dysphagia, regurgitation, nocturnal cough, intermittent wheezing, and episodic dyspnea. She also reported a prior episode of unexplained syncope that had required hospitalization several months earlier. She had previously been informed that her esophagus was markedly dilated in association with her gastric band and had undergone band adjustments.

Chest radiography obtained during the acute episode showed marked gaseous distension of the esophagus measuring up to 80 mm (Figure 1). Prior imaging demonstrated baseline severe esophageal dilatation. Contrast-enhanced computed tomography revealed an esophageal diameter of approximately 75 mm, with the dilated esophagus extending into the mediastinum and producing apparent mass effect on the trachea and left atrium (Figures 2A-2C). Subsequent imaging after gastric band deflation demonstrated substantially less esophageal dilatation (Figure 2D). The available imaging established severe esophageal enlargement and apparent adjacent mediastinal compression but did not, by itself, establish aperistalsis or a specific achalasia subtype.

**Figure 1:**
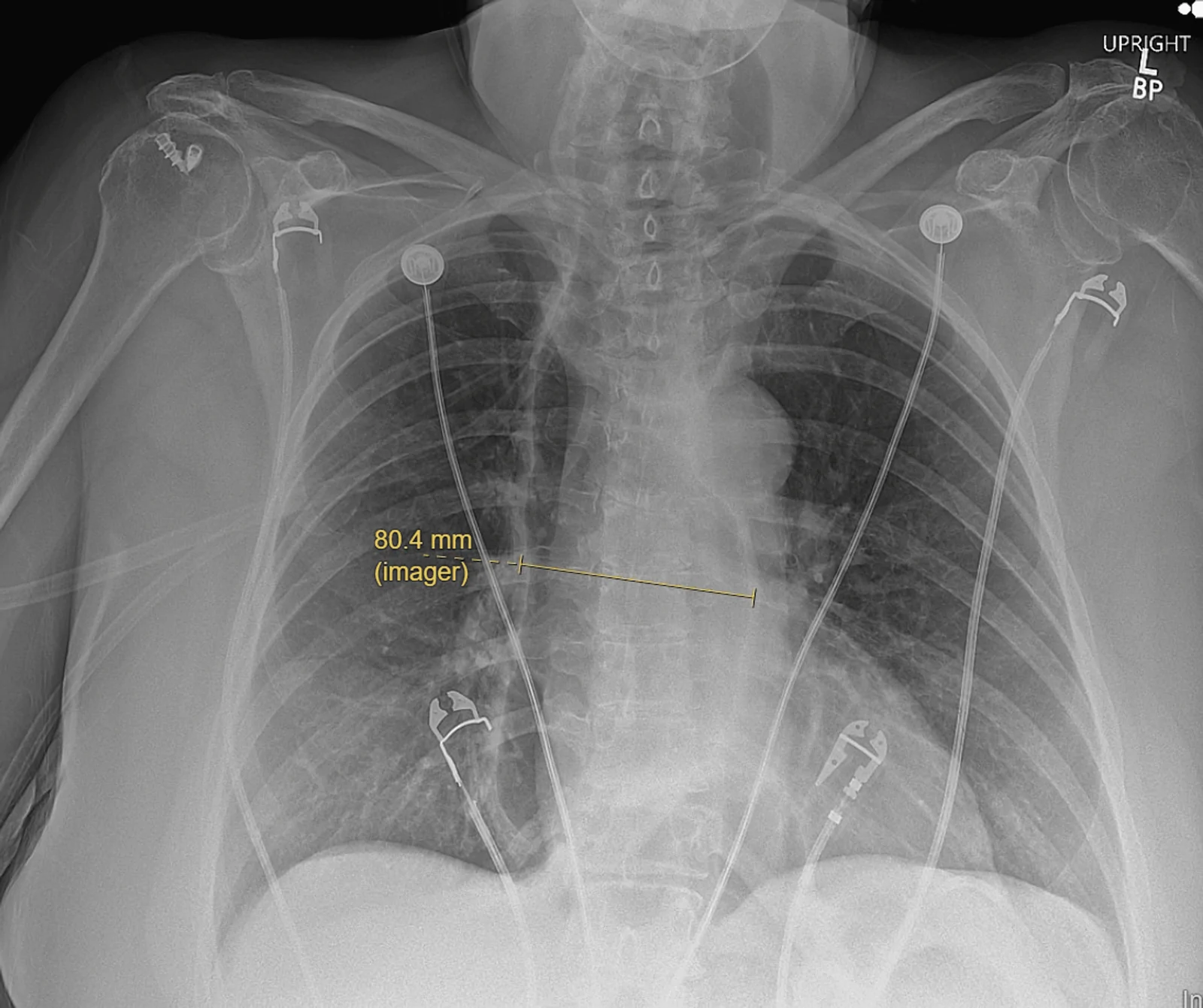
Chest radiograph obtained during the acute episode, showing marked gaseous distension of the esophagus, measuring 80.4 mm in the largest diameter.

**Figure 2:**
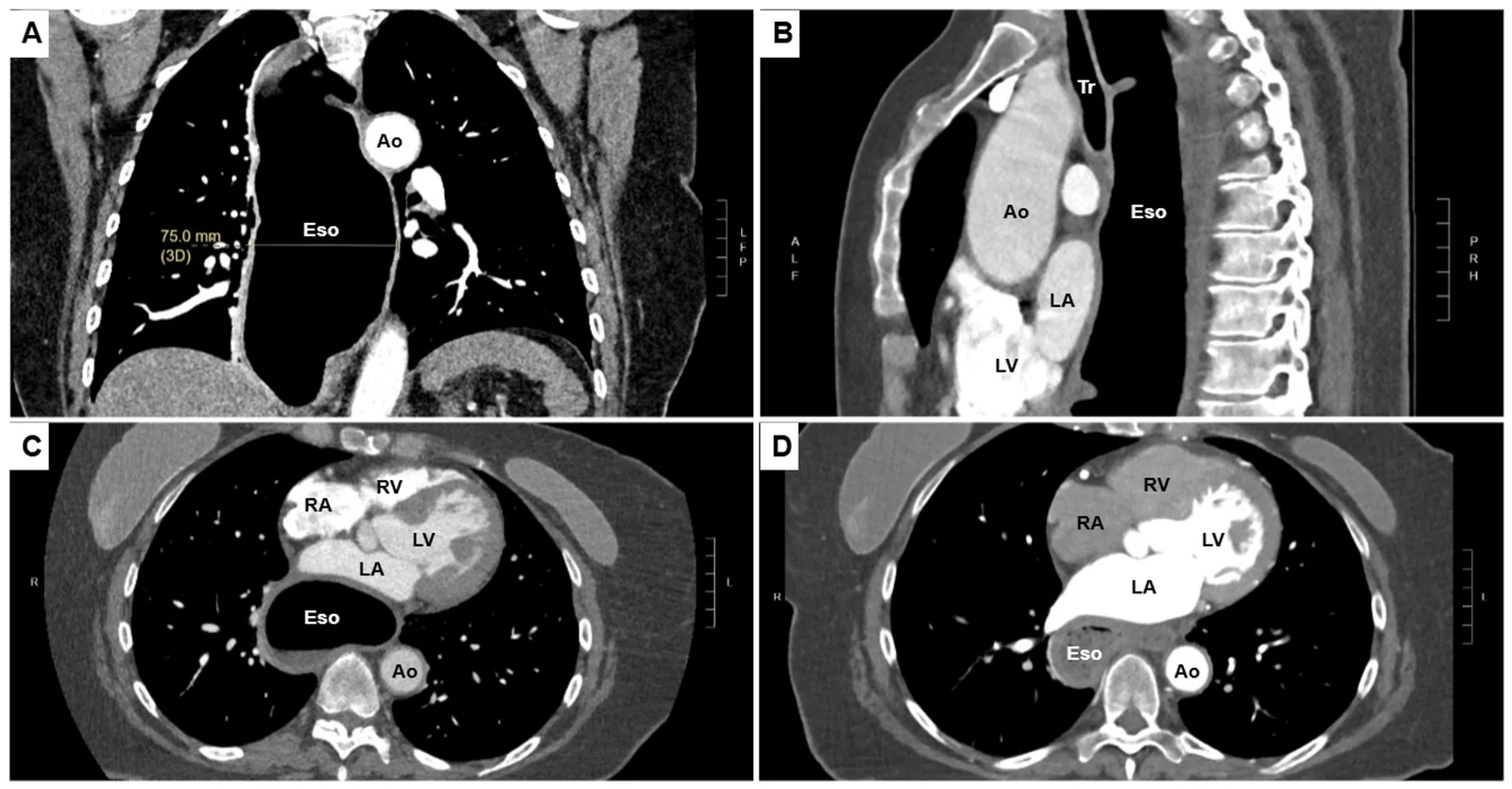
Prior contrast-enhanced CT of the chest. Coronal (A) and sagittal (B) reconstructions and axial images (C) demonstrate marked esophageal dilatation measuring up to 75 mm, with mass effect and compression of the trachea and left atrium. In comparison, the axial image (D) obtained after deflation of the gastric band demonstrates decompression of the esophagus with decreased mass effect on the left atrium. Eso: esophagus; Ao: aorta; Tr: trachea; LA: left atrium; LV: left ventricle; RA: right atrium; RV: right ventricle

Cardiac evaluation did not identify evidence of acute myocardial injury or decompensated heart failure. High-sensitivity troponin-T values were 6 and 8 ng/L (reference range, 0-9 ng/L), and B-type natriuretic peptide was 141 pg/mL (reference range, 0-900 pg/mL). Electrocardiography demonstrated sinus rhythm, with a rate of 92 beats/min initially and approximately 60 beats/min subsequently, without evidence of acute ischemia. There was no clinical evidence of acute heart failure.

The acute onset of symptoms immediately following ingestion of effervescent granules could strongly suggest rapid expansion of the already dilated esophagus. Transient compression of the trachea, potentially compounded by reflex airway narrowing and acute hypoventilation, provided a plausible explanation for her respiratory distress and hypercapnia. A similar diagnostic dilemma has been reported in patients with achalasia-associated megaesophagus, in whom acute airway compression initially mimicked anaphylaxis [4].

Following stabilization, the patient was admitted for observation. Because the clinical presentation was inconsistent with ongoing anaphylaxis, no additional treatment for anaphylaxis was administered. She was managed conservatively with aspiration precautions, avoidance of further gas-producing agents, and close clinical monitoring. Gastroenterology and surgery were consulted regarding definitive management of her severe esophageal dilatation and longstanding gastric band in the hospital. The patient elected to follow up with her outpatient surgery for band decompression given resolved acute respiratory distress.

The patient remained clinically stable without recurrent respiratory distress and was discharged with outpatient surgery and endocrinology follow-up on the following day. She was advised to avoid effervescent preparations and to consume liquids in small volumes. In the outpatient setting, the gastric band was subsequently deflated, after which her symptoms related to esophageal dilation improved substantially, although she experienced weight gain. Her outpatient endocrinologist subsequently initiated tirzepatide (Zepbound) for weight management, which she has continued.

## Discussion

This case illustrates a rare acute presentation of longstanding, severe esophageal dilatation following LAGB. The key clinical feature was abrupt respiratory distress occurring shortly after ingestion of an effervescent preparation in a patient with marked pre-existing esophageal dilatation and impaired esophageal emptying. Although the precise mechanism cannot be established from the available imaging, acute gaseous distension of the chronically dilated esophagus was suspected to have contributed to the respiratory event.

The pathophysiology of esophageal dilatation after LAGB is primarily related to chronic functional outflow obstruction at the gastroesophageal junction. Excessive band restriction increases resistance to esophageal emptying and may initially result in increased contractile activity. With persistent obstruction, however, esophageal peristalsis may become progressively impaired, leading to esophageal enlargement and an achalasia-like pattern of impaired clearance [1,3]. This process can develop years after band placement and may persist or progress despite the absence of prominent gastrointestinal symptoms.

The reported incidence of esophageal dilatation varies substantially across studies, reflecting differences in diagnostic criteria, study populations, and follow-up duration. Naef et al. reported esophageal dilatation in 40 of 157 patients (25.5%) during long-term follow-up, defining dilatation as an esophageal diameter of ≥35 mm; diameters in that cohort ranged from 35.0 to 94.6 mm [1]. de Jong et al. used a different definition, considering dilatation to be an increase in esophageal diameter to >130% of the postoperative diameter, and reported dilatation in 25 of 45 patients (55.6%) [2]. Le Page et al. reported radiographic esophageal dilatation in 7 of 50 patients (14%), whereas 17 patients (34%) had radiologic abnormalities encompassing dysmotility and/or dilatation [5]. These differences underscore the importance of distinguishing radiographic esophageal enlargement from esophageal dysmotility and from severe symptomatic megaesophagus. The prevalence of massive dilatation associated with clinically significant mediastinal compression is not well established.

In our patient, prior contrast-enhanced CT demonstrated an esophageal diameter of up to 75 mm, with marked enlargement extending into the mediastinum and apparent mass effect on the trachea and left atrium. This degree of dilatation is substantial but not unprecedented. Naef et al. reported esophageal diameters up to 94.6 mm [1], while Lea et al. described a 75-mm megaesophagus associated with severe tracheobronchial compression and an acute airway emergency following gastric banding [3]. Thus, the contribution of this case is not the absolute esophageal diameter or the presence of mediastinal compression alone, but rather the acute respiratory presentation occurring in close temporal association with ingestion of an effervescent preparation in a patient with severe pre-existing esophageal disease.

The prior CT provides evidence of chronic anatomic distortion but does not establish the degree of airway or cardiac compression during the acute episode. No contemporaneous CT or other dynamic imaging was obtained to demonstrate an interval increase in esophageal diameter or transient worsening of tracheal compression. Similarly, the observed left-atrial deformation does not establish hemodynamically significant cardiac compression. Although cardiac compression is a plausible consequence of severe esophageal enlargement, its functional significance in this patient remains uncertain. De Silva et al. reported a patient with longstanding LAGB-associated megaesophagus in whom CT and echocardiography demonstrated left-atrial compression that improved following band removal, supporting the potential clinical relevance of this anatomic relationship [6]. In the present case, however, the prior left-atrial mass effect should be regarded as an anatomically relevant finding rather than a demonstrated cause of the patient's prior dyspnea, wheezing, or syncope.

The acute respiratory event occurred shortly after ingestion of effervescent granules [7]. Effervescent preparations generate carbon dioxide through an acid-bicarbonate reaction in the presence of water. In a normally functioning esophagus, the generated gas is rapidly transmitted into the stomach. In a markedly dilated esophagus with impaired clearance, however, gas may transiently accumulate within the esophageal lumen and increase intraluminal volume. In a patient whose esophagus already occupies substantial mediastinal space, even transient additional distension could potentially exacerbate pre-existing mass effect on adjacent structures. This represents a plausible mechanism in our patient, although the absence of dynamic imaging prevents direct demonstration of transient worsening of airway compression.

Several features support a temporal relationship between the effervescent preparation and the acute respiratory distress. Symptoms developed within moments of ingestion, chest radiography obtained during the episode demonstrated marked gaseous distension of the esophagus, and the respiratory symptoms subsequently improved as the acute event resolved. Transient hypercapnia was also consistent with hypoventilation during the episode. However, these findings do not prove that the gaseous distension caused transient tracheal compression, nor do they definitively exclude an allergic mechanism. In particular, no serum tryptase was obtained during the event, limiting retrospective assessment for anaphylaxis. The appropriate interpretation is therefore that acute gaseous distension of the chronically dilated esophagus was a suspected contributor to the respiratory distress rather than a definitively established cause.

The presentation also highlights an important diagnostic challenge: severe esophageal dilatation can produce respiratory symptoms that resemble anaphylaxis. Sudden wheezing and respiratory distress following ingestion of a substance appropriately raise concern for an allergic reaction. However, in a patient with known severe esophageal dilatation, structural causes of respiratory compromise should also be considered, particularly when the presentation lacks other characteristic features of anaphylaxis. The absence of cutaneous manifestations, hypotension, angioedema, or persistent bronchospasm may support consideration of alternative diagnoses, although their absence alone does not exclude anaphylaxis. Herrin et al. described a patient with achalasia-related megaesophagus who was initially treated for anaphylaxis before imaging demonstrated tracheal compression by the dilated esophagus [4]. Similarly, Miyamoto et al. reported acute airway obstruction associated with megaesophagus that improved following esophageal decompression [8]. These reports support the broader concept that massive esophageal dilatation can produce acute respiratory compromise, while the present case adds the potential role of acute gaseous distension following an effervescent preparation.

Management of severe esophageal dilatation after LAGB depends on the degree of obstruction, symptoms, and reversibility of the esophageal dysfunction. Band deflation is generally an initial therapeutic measure because reducing band restriction may decrease gastroesophageal outflow resistance. However, severe or longstanding dilatation may persist despite deflation and may ultimately require band removal. Arias et al. reported resolution of severe esophageal dilatation following removal of the gastric band in patients who failed to improve with conservative measures [9]. Ulmer et al. similarly described patients with advanced esophageal dilatation in whom band deflation was insufficient and surgical intervention was required [10]. These observations emphasize the importance of recognizing progressive esophageal dysfunction before irreversible changes develop.

The present case also underscores the importance of chronic symptoms in patients with longstanding gastric bands. Dysphagia, regurgitation, nocturnal cough, wheezing, and unexplained dyspnea may reflect progressive esophageal dysfunction rather than reflux or primary pulmonary disease alone. Recognition of these symptoms may provide an opportunity to evaluate esophageal emptying and band-related obstruction before severe megaesophagus and mediastinal compression develop.

This case has several limitations. First, the 75-mm esophageal diameter and associated tracheal and left-atrial mass effect were demonstrated on prior CT and therefore represent pre-existing anatomy rather than measurements obtained during the acute respiratory event. The exact interval between the prior CT and the acute presentation was not available, and no serial imaging was obtained to demonstrate interval enlargement or dynamic changes in airway caliber. The acute event was evaluated with chest radiography, which demonstrated marked gaseous esophageal distension to 80.4 mm but did not permit quantitative assessment of tracheal compression comparable to CT. Second, dynamic imaging was not feasible during the acute episode, limiting our ability to establish the proposed mechanical mechanism. Third, serum tryptase was not obtained, and therefore an allergic mechanism cannot be definitively excluded retrospectively. Finally, although transient hypercapnia was compatible with hypoventilation, it is not specific for the mechanism of respiratory distress.

Taken together, the findings support a model in which severe chronic esophageal dilatation created a vulnerable mediastinal anatomy, while ingestion of an effervescent preparation may have produced acute gaseous distension that contributed to transient respiratory compromise. The temporal association, acute gaseous esophageal distension on radiography, transient hypercapnia, and subsequent clinical improvement make this mechanism plausible, but the absence of contemporaneous dynamic imaging requires that it remain a suspected rather than proven explanation. Clinicians evaluating patients with longstanding LAGB and severe esophageal dilatation should therefore be aware that acute respiratory distress after ingestion of a gas-generating preparation may have an esophageal component and should consider this possibility alongside anaphylaxis and other causes of acute respiratory compromise.

## Conclusions

Severe esophageal dilatation is an important long-term complication of LAGB that may cause substantial mediastinal mass effect. In this patient, acute respiratory distress immediately after an effervescent preparation was consistent with acute gaseous esophageal expansion contributing to respiratory compromise. However, direct dynamic airway compression was not demonstrated, and anaphylaxis could not be definitively excluded. This case highlights the importance of assessing esophageal emptying and gastric-band status and carefully considering precautions for gas-producing agents in patients with severe esophageal dilatation.
